# Genomic analysis and replication kinetics of the closely related EHV-1 neuropathogenic 21P40 and abortigenic 97P70 strains

**DOI:** 10.1186/s13567-024-01434-3

**Published:** 2025-01-13

**Authors:** Eslam Mohamed, Ines Zarak, Nick Vereecke, Sebastiaan Theuns, Kathlyn Laval, Hans Nauwynck

**Affiliations:** 1 Department of Translational Physiology, Infectiology and Public Health, Faculty of Veterinary Medicine, Ghent University, 9820 Merelbeke, Belgium; 2 Department of Animal Medicine, Faculty of Veterinary Medicine, Benha University, Moshtohor, 13736 Egypt; 3 PathoSense BV, Pastoriestraat 10, 2500 Lier, Belgium

**Keywords:** EHV-1, EHM, respiratory mucosa, vaginal mucosa, monocytes, ICP22

## Abstract

Varicellovirus equidalpha 1, formerly known as Equid alphaherpesvirus 1 (EHV-1), is highly prevalent and can lead to various problems, such as respiratory problems, abortion, neonatal foal death, and neurological disorders. The latter is known as equine herpes myeloencephalopathy (EHM). Cases of EHM have significantly increased since the beginning of the twenty-first century. The genomic sequences of five isolates associated with the fatal neurological outbreak in Valencia, Spain, in 2021 were analyzed and documented. The genome and replication kinetics of the Belgian EHM isolate 21P40, associated with the Valencia outbreak, and the well-characterized abortigenic strain 97P70 were compared. Both strains exhibited a nucleotide identity of 99.96%, with only seven genetic mutations in ORFs 13, 24, 30, 32, 40, 65, and 71. Isoleucine and asparagine at loci 291 and 207 of ORF30 (DNA polymerase) and ORF65 (ICP22), respectively, were unique to isolates from the Valencia outbreak. The replication kinetics of these two genetically closely related strains were determined in rabbit kidney (RK-13), equine respiratory, and vaginal mucosal explant cells, as well as equine blood monocytes (CD172a^+^). Both strains replicated equally well in RK-13 cells. The neuropathogenic isolate 21P40 exhibited a more extensive infection in respiratory explants and blood monocytes, as demonstrated by more plaques and single infected leukocytes, and a higher percentage of infected monocytes. In contrast, vaginal explants infected with the abortigenic strain 97P70 demonstrated more plaques and single infected leukocytes. In conclusion, 21P40 replicated significantly different compared to 97P70 but shared similarities with the Belgian well-studied neuropathogenic EHV-1 strain 03P37.

**Supplementary Information:**

The online version contains supplementary material available at 10.1186/s13567-024-01434-3.

## Introduction

Varicellovirus equidalpha 1, formerly known as Equid alphaherpesvirus 1 (EHV-1), belongs to the subfamily *Alphaherpesvirinae* causing primarily infections of the upper respiratory tract of horses. The genome of EHV-1 consists of a single linear double-stranded DNA of approximately 150 kbp and comprises unique long (U_L_) and short (U_S_) regions flanked by identical internal (IR) and terminal (TR) repeats. It carries at least 76 open reading frames (ORFs), of which ORF30 encodes for the DNA polymerase, ORF64 encodes for the immediate early regulatory protein ICP4, ORFs 5, 63, 65, and 77 encode for the early regulatory proteins ICP27, ICP0, ICP22, and IR2, respectively, and ORF12 encodes for the late regulatory protein VP16 [1–8].

The most important impact of EHV-1 infection is the manifestation of respiratory problems in young horses. However, it can also result in abortions, premature neonatal mortality in foals, and a neurological condition referred to as equine herpesvirus myeloencephalopathy (EHM) [9, 10]. The emergence of EHM outbreaks can result in severe economic losses and have negative consequences for the equine industry, as evidenced by recent worldwide outbreaks [11–14].

During and after the International Valencia Spring Tour held in Spain in February 2021, an EHM outbreak of significant severity was recorded, making it one of the most severe outbreaks in Europe so far. The event in question showcased a total of 752 horses. A total of 18 dead horses were reported and confirmed to be associated with cases in ten countries, including Belgium, Denmark, France, Germany, Italy, Qatar, Spain, Slovakia, Sweden, and Switzerland, in which quarantine regulations were immediately implemented to prevent further dissemination [15].

The occurrence of EHM has been suggested to be associated with a single nucleotide polymorphism (SNP) located at position 2254 of ORF30, encoding the DNA polymerase. This SNP results in the substitution of the original asparagine (N) with aspartic acid (D) at position 752 within the DNA polymerase. Still, previous studies, conducted in several countries, showed a big variability (52 to 97%), linking the N_752_ (A_2254_) marker to abortion cases [13, 16–21].

Five EHV-1 isolates from horses in Belgium and France, linked to the neurological outbreak during the Valencia tour in 2021, were sequenced and reported in May 2021. All strains exhibited the H250/N752/Y753/K990 genotype of the DNA polymerase and were closely related to each other and phylogenetically grouped into clade 10. In addition to those neuropathogenic isolates, clade 10 contains four sequenced abortigenic isolates, including the well-characterized Belgian abortigenic 97P70 strain [13].

This study aimed to conduct a thorough comparative analysis of the available EHV-1 genomes and replication kinetics of the Belgian neuropathogenic 21P40 strain from the Valencia outbreak and the abortigenic 97P70 strain.

## Materials and methods

### Viruses

Two Belgian EHV-1 strains were grown in RK-13 cells. The neuropathogenic strain 21P40 was originally isolated in 2021 from a nasal swab of a Belgian horse that had attended the Valencia Tour in February 2021 [13]. The abortigenic isolate 97P70 was first isolated from the lungs of an aborted fetus in 1997 [22].

### Genomic analysis

Both strains were sequenced at PathoSense BV using Oxford Nanopore Technology (ONT) on a MinION R.9.4 flow cell [13]. The downstream analysis involved pairwise identity determination and multiple-sequence alignment using the Basic Local Alignment Search Tool (BLAST v2.12.0 +) and Multiple Alignment using Fast Fourier Transform (MAFFT v7.471). The tools were executed with their *default* configurations. The comparative analysis of genomes encompassed all strains of EHV-1 (*n* = 127) that were accessible in June 2021 in the GenBank repository and previous studies.

### Tissue samples and cells

#### Respiratory and vaginal explants

Samples of tissues were collected from three healthy horses post-slaughter, specifically from the deep intranasal septum, the proximal segment of the trachea, and the middle portion of the vagina. The tissues were submerged in transport medium consisting of 500 mL of phosphate-buffered saline (PBS) containing calcium and magnesium, supplemented with 100 U/mL penicillin (ThermoFisher Scientific, Paisley, UK), 0.1 mg/mL streptomycin (ThermoFisher Scientific), 0.1 mg/mL gentamicin (ThermoFisher Scientific), 0.1 mg/mL kanamycin (Merck, Darmstadt, Germany), and 0.25 μg/mL amphotericin B (ThermoFisher Scientific) and transported to the laboratory on ice.

Upon arrival, the mucosae were separated from the underlying tissues and divided into uniform explants measuring 25 mm^2^ using sterile tweezers and surgical blades No. 24 (Swann-Morton, Sheffield, England). To evaluate the viability of the mucosal explants, a single explant per tissue type was embedded in methylcellulose 2% (Methocel®; Sigma-Aldrich, St. Louis, USA) and snap-frozen using dry ice and ethanol 100%. The explants were subjected to an air–liquid interface cultivation method for 18 h at 37 °C with 5% CO_2_, as previously described by Vandekerckhove et al. in 2009 for the respiratory tissues [23] and Negussie et al. in 2016 for the vagina [24]. Briefly, explants were positioned onto sterilized fine-meshed gauzes with the epithelium facing upwards. The gauzes were then placed in 6-well plates containing serum-free medium, composed of a 50% mixture of Dulbecco’s modified eagle medium including Glutamax (DMEM; ThermoFisher Scientific) and Roswell Park Memorial Institute medium including Glutamax and HEPES (RPMI; ThermoFisher Scientific), supplemented with 100 U/mL penicillin, 0.1 mg/mL streptomycin, 0.1 mg/mL gentamicin and 0.25 μg/mL amphotericin B. To mimic the air–liquid interface, the medium covered the sides of explants, leaving the epithelium exposed to the air.

The ciliary beating of the epithelial cells of the nasal and tracheal explants was assessed by an Olympus IX50 light microscope, along with an in-situ cell death detection kit (Roche Diagnostics Corporation, Basel, Switzerland). This kit, that relies on the TUNEL technology, allowed to determine the number of apoptotic cells in the epithelium and lamina propria of all explants, which is related to the tissue viability.

#### Blood monocytes (CD172a^+^)

Twenty milliliters of blood were obtained from three horses through jugular venipuncture and collected into heparin 15 U/mL (Leo, Zaventem, Belgium). The samples were diluted with an equivalent volume of Dulbecco’s phosphate-buffered saline (DPBS) without calcium and magnesium (Gibco, Invitrogen, Paisley, UK). The isolation of peripheral blood mononuclear cells (PBMCs) was carried out through density centrifugation on Ficoll-Paque (density = 1.077 g mL^−1^) (GE Healthcare, Life Sciences) at 800 × *g* for 30 min at 18 °C. The PBMCs were obtained from the interphase and subjected to three rounds of washing with DPBS. The cells were suspended in leukocyte medium (LM), composed of RPMI, supplemented with 5% fetal calf serum (FCS, Gibco), 0.1 mM non-essential amino acids (NEAA, Gibco), 1 mM sodium pyruvate (Gibco), 100 U/mL penicillin, 0.1 mg/mL streptomycin, 0.1 mg/mL gentamicin, and 0.25 μg/mL amphotericin B. Subsequently, the cells were cultured on inserts pre-coated with 500 µL of 0.47 mM Poly-L-Lysine (Sigma Aldrich) and placed in 24-well plates (Nunc A/S, Roskilde, Denmark) at a density of one million cells per mL and maintained at 37 °C with 5% CO_2_. After 12 h, adherent monocytic cells were washed three times with RPMI to eliminate nonadherent lymphocytes.

#### Rabbit kidney (RK-13) cells

Rabbit kidney (RK-13) cells on inserts (0.47 mM Poly-L-Lysine) in 24-well plates were used as a control culture in this study. They were maintained in RK-13 medium, composed of Modified Eagle's medium (MEM, ThermoFisher), supplemented with 5% FCS, 100 U/mL penicillin, 0.1 mg/mL streptomycin, 0.1 mg/mL gentamicin, and 0.25 μg/mL amphotericin B.

### Virus inoculation

To study the replication kinetics, three biological replicates per tissue and cell type were used. The virus stocks used for virus inoculation were at the third and sixth passage for EHV-1 21P40 and EHV-1 97P70, respectively.

#### Respiratory and vaginal explants

Following overnight cultivation, the explants were inoculated in a 24-well plate by submerging them in 1 mL of inoculum containing 10^6.5^ TCID_50_ of EHV-1 21P40 or 97P70 and maintained at 37 °C and 5% CO_2_ for 1 h. Following two rounds of washing with a warm serum-free medium, the explants were repositioned onto their respective gauzes. At 1, 24, and 48 h post-inoculation (hpi), the medium of the explants was collected for extracellular virus titration. Subsequently, the explants were embedded in Methocel®, snap-frozen, and stored at -70 °C. Mock-inoculated explants with only serum-free medium were conducted concurrently.

#### Blood monocytes (CD172a^+^) and RK-13 cells

Adherent blood monocytes and RK-13 cells were exposed to 200 µL of EHV-1 21P40 or EHV-1 97P70 in a 24-well plate; two wells per time point, at a multiplicity of infection (MOI) of 5 for 1 h at 37 °C and 5% CO_2_. The blood monocytes and RK-13 cells were washed twice with a warm respective medium to remove the viral inoculum. At 1, 12, 24, and 48 hpi, cells from the first well were fixed in 100% methanol at  -20 °C for 20 min to assess the quantity and percentage of EHV-1 infected cells through immunofluorescent staining. Moreover, the second well’s supernatant and cells were collected for extracellular and intracellular virus titration, respectively. Mock-infected cells with only the respective medium were processed in parallel.

### Replication kinetics

#### Respiratory and vaginal explants

##### Plaque analysis and penetration through the basement membrane

At corresponding time points, 50 cryosections, each measuring 16 µm, were cut from explants and subsequently fixed in 100% methanol at -20 °C for 20 min. Firstly, endogenous biotin was blocked using the Avidin/Biotin Blocking Kit (Invitrogen). The basement membrane was subsequently stained using monoclonal antibodies (mAb) against mouse anti-collagen VII 1/300 (Sigma-Aldrich), followed by incubation in secondary Texas Red®-labelled goat anti-mouse IgG antibodies 1/100 (Molecular Probes, Eugene). Subsequently, the viral proteins were stained with biotinylated equine polyclonal anti-EHV-1 IgG 0.15 mg/mL, previously prepared in our laboratory [25] (1 h at 37 °C) and streptavidin FITC® 1/200 (Molecular Probes; 1 h at 37 °C). Between the two staining steps and at the end, the cryosections were washed three times with PBS. Finally, the cryosections were mounted with glycerin DABCO (Janssen Pharmaceutica, Beerse, Belgium). The stained cryosections were analyzed using a Leica TCS SP2 laser scanning spectral confocal system (Leica Microsystems, GmbH, Wetzlar, Germany), using an argon 488-nm laser line and a Gre/Ne 543-nm laser line to excite FITC and Texas red, respectively.

##### Quantification and identification of single EHV-1-infected cells

The number and identity of single EHV-1-infected epithelial cells and leukocytes in the epithelium and the lamina propria were assessed by a double immunofluorescence staining. At corresponding time points, 40 cryosections of 16 µm were prepared and fixed in 100% methanol for 20 min at -20 °C. Twenty cryosections were stained for each cell-surface marker separately. In the first step, the mAb DH59B IgG1 1/100 (VMRD Inc., Pullman, USA) and UC F6G-3 IgG1 1/50 (University of California, Davis, USA) were used as markers for CD172a^+^ cells from the monocytic lineage or CD3^+^ cells (pan T-lymphocytes), respectively. Subsequently, cryosections were incubated with secondary Texas Red®-labelled goat anti-mouse antibodies 1/100. In the second step, all cryosections were stained for viral proteins by incubation of biotinylated equine polyclonal anti-EHV-1 IgG 0.15 mg/mL, followed by streptavidin FITC® 1/200. Isotype-corresponding control (IgG1) mouse monoclonal anti-PRV gD antibody 13D12 was included [26]. In general, antibodies were incubated for 1 h at 37 °C. Finally, cryosections were washed three times in PBS and mounted with glycerin-DABCO. The number of individual infected cells and their identity were determined by confocal laser-scanning microscopy. EHV-1 infected cells were counted in five randomly chosen fields of 100 cells.

##### Virus titration

At each time point, serum-free medium from each well was collected and centrifuged at 400 × *g* for 10 min at 4 °C and the supernatant was stored at -70 °C. Titration was performed on RK-13 cells and incubated at 37 °C and 5% CO_2_ and monitored for 7 days. The final virus titer was calculated as 50% tissue culture infective dose (TCID_50_) according to the Reed and Muench formula (1938) [27].

#### Blood monocytes (CD172a^+^) and RK-13 cells

##### Percentage of EHV-1-infected cells

Inserts with adherent blood monocytes and RK-13 cells were fixed in 100% methanol at -20 °C for 20 min. In the first step, mAb DH59B 1/100 was used as a marker for CD172a^+^ cells. Subsequently, inserts were incubated with secondary Texas Red®-labelled goat anti-mouse IgG antibodies 1/100. In a second step, inserts were stained for viral proteins by incubation with biotinylated equine polyclonal anti-EHV-1 IgG 0.15 mg/mL, followed by streptavidin-FITC® 1/200. In all steps, antibodies were incubated for 1 h at 37 °C. Finally, inserts were washed three times in PBS and mounted with glycerine-DABCO. The EHV-1-infected blood monocytes and RK-13 were detected, and their percentage was assessed by confocal laser scanning microscopy.

##### Extracellular and intracellular virus titration

At corresponding sample collection times (1, 12, 24, 48 hpi), both extracellular and intracellular virus titers were determined as described above. The supernatant, containing extracellular virus, was collected, and centrifuged at 400 × *g* for 10 min at 4 °C prior to storage at -70 °C. Cells containing the intracellular virus were harvested by flushing and scraping the cells in fresh 1 mL of respective cell medium. Afterwards, they were centrifuged at 400 × *g* for 10 min at 4 °C. The cells were lysed through three cycles of freeze-thawing and then stored at -70 °C. Viral titrations were conducted on RK-13 cells as described previously.

### Statistical analysis

For statistical significance, the data were evaluated by ordinary two-way analysis of variance (ANOVA) with a post hoc Tukey’s multiple comparisons test. When *p*-values were ≤ 0.05, the differences in the results were considered significant. The statistical analysis of the data was done using Prism 9 for macOS, version 9.3.0.

## Results

### Genomic analysis

Pairwise identity determination revealed a high nucleotide identity between the abortigenic EHV-1 97P70 and neurovirulent EHV-1 21P40 strain (99.96%) with 98% query coverage. The sequence alignment showed differences in seven ORFs, including five SNPs in ORFs 13, 30, 32, 40, 65, one deletion in ORF24 and one insertion in ORF71 (Table 1).

**Table 1:** **Nucleotide and amino acid substitutions in EHV-1 97P70 and 21P40**

ORF	Protein	Nucleotide position	Amino acid position	EHV-1^a^ 97P70	EHV-1^a^ 21P40
13	Tegument protein VP13/14	927	309	GCA (Alanine)	GCG (Alanine)
24	Large tegument protein	Deletion		10,431 nucleotides (3477 codons)	10,374 nucleotides (3458 codons)
30	DNA polymerase catalytic subunit	872	291	AGC (Serine)	ATC (Isoleucine)
32	DNA packaging terminase subunit 2	539	180	ATC (Isoleucine)	ACC (Threonine)
40	Tegument protein	773	258	TAC (Tyrosine)	TGC (Cysteine)
65^b^	Regulatory protein ICP22	619	207	GAC (Aspartic acid)	AAC (Asparagine)
71	Envelope glycoprotein J (gp2)	Insertion		2400 (800 codons)	2415 (805 codons)

^a^Underlined letter is the changed nucleotide. Amino acid residue between brackets.

^b^This mutation occurred in both inverted and terminal repeats (IR and TR).

Upon the inclusion of other EHV-1 isolates available in the GenBank repository (*n* = 127 of which 28 are complete genomes) in the analysis, an amino acid substitution of the isoleucine at position 291 of ORF30 (DNA polymerase) and the asparagine at position 207 of ORF65 (regulator protein ICP22) were unique to the isolates from the Valencia neurological outbreak in 2021, including the 21P40 isolate. On the contrary, the isoleucine at position 180 of ORF32 (DNA packaging terminase subunit 2 ICP18.5) and tyrosine at position 258 of ORF40 (tegument protein) were unique for the abortigenic EHV-1 97P70 strain. The nucleotide substitution at position 927 of ORF13 (Tegument protein VP13/14) represents a silent mutation at the amino acid level at position 309. For ORF24 and 71, also structural variations (insertions/deletions) within their coding sequence were identified among isolates. In Additional file 1, a detailed analysis of the four single-point amino acid substitutions reported in EHV-1 97P70 and 21P40, in addition to locus 752 of the DNA polymerase, compared to other EHV-1 strains in the GenBank repository can be found.

### Replication kinetics in respiratory and vaginal explants

**Figure 1:**
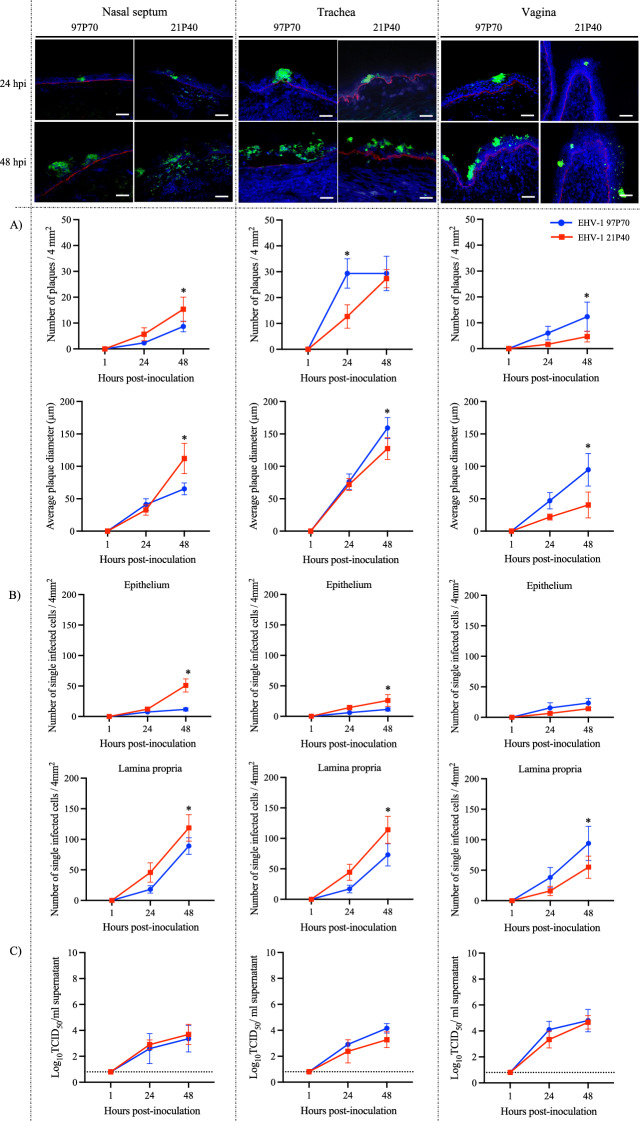
**Replication kinetics in the respiratory and vaginal mucosa. Representative confocal microscopy images of nasal septum, trachea and vagina infected with EHV-1 abortigenic 97P70 and neuropathogenic 21P40 at 1, 24 and 48 hpi (viral proteins in green and basement membrane in red)** **A** Plaque number and average plaque diameter. **B** Number of single infected cells **C** Virus titer. Data are presented as means of three experiments. Asterisk represents statistically significant difference. Scale bar 50 µm. Dashed line represents the limit of detection (LOD).

The replication of both strains progressed in a plaque-wise manner, spreading laterally. Plaques were detectable from 24 hpi. The plaques induced by both strains increased in number and size but did not traverse the basement membrane at all observed time points (Figure 1A).

In the nasal septum mucosa, plaques induced by both strains appeared from 24 hpi and increased at 48 hpi (Figure 1A left). The neuropathogenic EHV-1 21P40 exhibited more plaques at both time points with a significant difference at 48 hpi compared to the abortigenic EHV-1 97P70 (6 ± 3 and 15 ± 5 plaques for EHV-1 21P40 and 2 ± 1 and 9 ± 2 plaques for EHV-1 97P70 at 24 and 48 hpi, respectively). The plaque diameter increased over time for both strains. The abortigenic EHV-1 97P70 strain showed slightly but not significantly bigger (*p* = 0.77) plaques at 24 hpi (41.00 ± 8.89 µm and 32.73 ± 7.92 µm for EHV-1 97P70 and 21P40, respectively). However, at 48 hpi, the neuropathogenic EHV-1 21P40-induced plaques were significantly bigger (112.10 ± 23.29 µm and 65.20 ± 9.18 µm for EHV-1 21P40 and 97P70, respectively). Single infected cells were visible in the epithelium and lamina propria for both strains from 24 hpi and increased over time (Figure 1B left and Table 2). Both strains infected more CD172a^+^ monocytic cells in the epithelium than CD3^+^ cells. In the lamina propria, the neurovirulent EHV-1 21P40 strain infected more CD172a^+^ monocytic cells than CD3^+^ cells, while the abortigenic EHV-1 97P70 infected more CD3^+^ cells than CD172a^+^ monocytic cells. The titer of both strains showed a steady increase over time with no statistically significant differences between the two strains (Figure 1C left).

**Table 2:** **Percentage of single EHV-1 infected cells identified as CD172a**^**+**^** or CD3**^**+**^

Tissue	Region	CD172a^+^				CD3^+^			
		24 hpi		48 hpi		24 hpi		48 hpi	
		EHV-1 97P70	EHV-1 21P40	EHV-1 97P70	EHV-1 21P40	EHV-1 97P70	EHV-1 21P40	EHV-1 97P70	EHV-1 21P40
Nasal septum									
	Epithelium	28.9 ± 4.2	27.3 ± 6.8	33.9 ± 5.7	32.8 ± 7.9	20.6 ± 4.2	9.3 ± 8.5	23.4 ± 6.1	20.0 ± 8.8
	Lamina propria	31.9 ± 6.4	54.5 ± 10.8	24.5 ± 4.7	63.0 ± 9.0	38.7 ± 4.9	23.0 ± 9.9	33.4 ± 3.8	23.4 ± 4.6
Trachea									
	Epithelium	26.1 ± 6.7	29.1 ± 9.6	25.7 ± 15.1	28.4 ± 12.4	23.3 ± 8.9	10.3 ± 9.0	21.7 ± 2.9	18.7 ± 5.6
	Lamina propria	30.0 ± 8.7	58.5 ± 12.2	28.9 ± 8.8	59.9 ± 11.1	39.5 ± 10.7	23.6 ± 10.8	37.3 ± 13.4	24.9 ± 9.2
Vagina									
	Epithelium	28.9 ± 11.3	28.9 ± 7.7	29.9 ± 9.2	31.7 ± 2.7	20.2 ± 6.7	23.3 ± 8.8	23.3 ± 4.6	24.9 ± 7.7
	Lamina propria	45.9 ± 8.4	42.5 ± 6.6	46.4 ± 7.6	46.3 ± 6.7	36.1 ± 10.0	37.4 ± 14.1	40.7 ± 10.3	50.7 ± 6.2

Identity and percentage of individual EHV-1 infected cells were assessed in 20 sections per cell marker (CD172a^+^ and CD3^+^) in the epithelium and lamina propria of nasal septum, tracheal and vaginal explants infected with EHV-1 abortigenic 97P70 and neuropathogenic 21P40 at 24 and 48 hpi.

In the tracheal mucosa, plaques were observed at 24 hpi for both strains and increased over time (Figure 1A middle). Interestingly, at 24 hpi, the number of EHV-1 97P70 plaques was over two-fold the number of plaques induced by EHV-1 21P40 (29 ± 6 and 13 ± 5 plaques for EHV-1 97P70 and 21P40, respectively). However, at 48 hpi, the EHV-1 21P40 plaques number increased twice to nearly the same number of EHV-1 97P70 plaques, which remained constant (29 ± 7 and 27 ± 4 plaques for EHV-1 97P70 and 21P40). The average plaque diameter increased over time for both strains. EHV-1 97P70 plaques were significantly bigger at 48 hpi (76.20 ± 12.03 µm and 159.17 ± 16.00 µm for EHV-1 97P70 and 71.93 ± 9.25 µm and 127.40 ± 16.75 µm for EHV-1 21P40 at 24 and 48 hpi, respectively). Single infected cells were present in the epithelium and lamina propria for both strains from 24 hpi and increased at 48 hpi (Figure 1B middle and Table 2). The behavior of both strains in the epithelium and the lamina propria was similar to their behavior in the nasal septum. The titer of both strains increased over time, but no statistical significance was observed between the two strains (Figure 1C middle).

In the vaginal mucosa (Figure 1A right), both strains induced plaques at 24 hpi which increased over time. The abortigenic EHV-1 97P70 showed a better replication with over two-fold difference in the number and size of induced plaques (6 ± 3 and 12 ± 6 plaques of 46.90 ± 12.64 µm and 94.77 ± 25.13 µm at 24 and 48 hpi, respectively), with a significant difference at 48 hpi compared to EHV-1 21P40 (2 ± 1 and 5 ± 2 plaques of 21.80 ± 4.56 µm and 40.36 ± 19.96 µm at 24 and 48 hpi, respectively). Both strains infected single cells in the epithelium and lamina propria at 24 hpi and increased at 48 hpi (Figure 1B right and Table 2). More infected CD172a^+^ monocytic cells than CD3^+^ cells in the epithelium and lamina propria were observed in both strains. Like the respiratory mucosa, the titer of both strains increased over time with no significant difference (Figure 1C right).

### Replication kinetics in blood monocytes (CD172a^+^) and RK-13 cells

**Figure 2:**
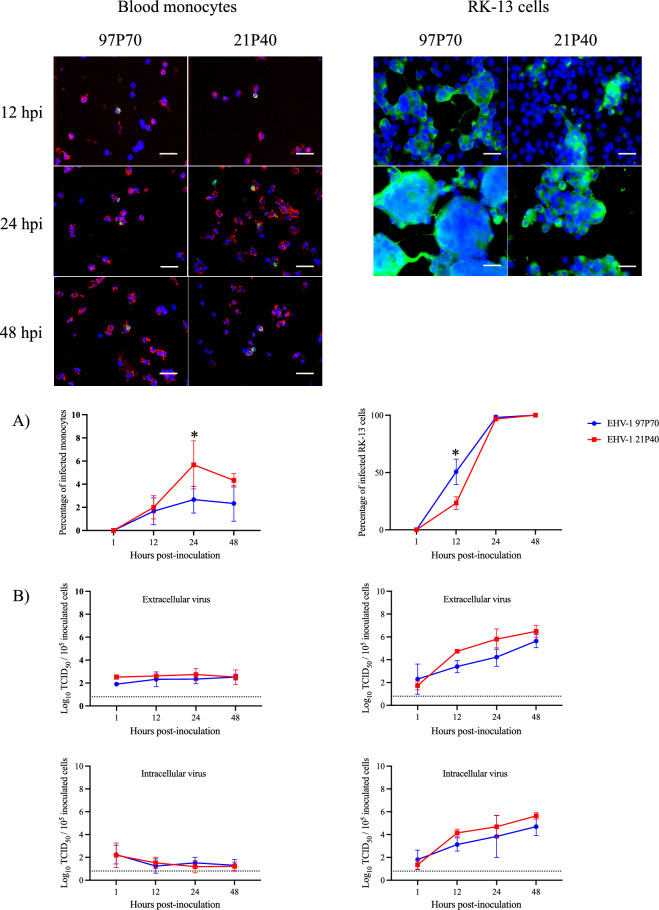
**Replication kinetics in blood monocytes and RK-13 cells. Representative confocal microscopy images of blood monocytes and RK-13 cells infected with EHV-1 abortigenic 97P70 and neuropathogenic 21P40 at 1, 12, 24 and 48 hpi (viral proteins in green and CD172a marker in red)**. **A** Percentage of infected cells. **B** Extracellular and intracellular virus titers. Data are presented as means of three experiments. Values with an asterisk are statistically different. At 48 hpi, RK-13 cells were detached. Scale bar 25 μm. Dashed line represents the limit of detection (LOD).

The replication of both strains in blood monocytes was restricted compared to RK-13 cells (Figure 2). The proportion of infected monocytes (Figure 2A) exhibited a temporal pattern whereby it rose steadily, reaching its maximum at 24 hpi, and subsequently declined at 48 hpi. At 12 hpi, both strains exhibited a similar level of infection (2 ± 1% for EHV-1 21P40 and 2 ± 1% for EHV-1 97P70). However, at 24 and 48 hpi, the neuropathogenic 21P40 isolate replicated better in comparison to the abortigenic 97P70 strain (6 ± 2% and 4 ± 1% for EHV-1 21P40 and 3 ± 1% and 2 ± 1% for EHV-1 97P70 at 24 and 48 hpi, respectively) with a statistically significant difference at 24 hpi. On the other hand, the percentage of EHV-1 97P70-infected RK-13 cells showed a steady increase till it reached 98 ± 2% at 24 hpi, while the percentage of EHV-1 21P40-infected RK-13 cells surged from 23 ± 6 to 97 ± 2% at 12 and 24 hpi, respectively. The significant difference between both strains was observed at 12 hpi (51 ± 11% for EHV-1 97P70 and 23 ± 6% for EHV-1 21P40). Throughout the experiment, the restricted replication behavior of both isolates in blood monocytes was consistent (Figure 2B), as manifested by the nearly constant intracellular and extracellular virus titer at different time points. In contrast, in RK-13 cells, the intracellular and extracellular virus titer steadily increased through the experiment time points.

## Discussion

Equine herpesvirus-1 (EHV-1) has drawn more attention in the past decade due to recurrent outbreaks of abortions and EHM in China, Europe, New Zealand, and the USA that have caused significant financial losses in the global equine industry. Although experimental and field studies have been conducted to understand the pathogenesis of these abortions and EHM, the mechanism by which the viral factors impact the clinical picture of EHV-1 infection is still poorly understood [11, 13, 28–36]. This work sheds new light on the viral factors that are presumably linked to the higher susceptibility to developing EHM.

Previously, a link between a unique SNP in the catalytic subunit of the viral DNA polymerase (ORF30) and EHV-1 outcome had been reported. It was suggested that strains encoding D_752_ were associated with neurological outbreaks, while strains carrying N_752_ were more likely linked with non-neurological cases [16, 37, 38]. Surprisingly, this single amino acid substitution (N752D), located in the palm region of the DNA polymerase (ORF30), was not reported in the five strains isolated from the Valencia outbreak and documented in the study of Vereecke et al. [13]. Here, our Valencia strains (including the Belgian 21P40 isolate from the Valencia outbreak) showed a genotype with an isoleucine at position 291 and not the serine within the N-terminal domain of the DNA polymerase (ORF30), which guides the single-stranded template to the polymerase active site [39]. Similar findings were documented in the examination of ORF30 among isolates associated with neurological outbreaks in the United Kingdom between 2012 and 2013, where solely the H250R mutation in the N-terminal domain was identified [38]. Furthermore, it is noteworthy that the EHV-1 isolates identified in an outbreak characterized by a combination of EHM and abortion cases in Germany in 2012 exhibited the G_2254_/D_752_ DNA polymerase genotype [33]. A novel EHV-1 DNA polymerase genotype C_2254_/H_752_ was identified by Sutton et al*.* during an outbreak that was documented in France in 2018 and Pusterla et al. during an investigation of EHV-1 isolates from 2019 and 2022 in the USA [39, 40]. These studies confirm that the N752D polymorphism in ORF30 does not exclusively serve as a neuropathogenic marker. Other mutations in the DNA polymerase, other ORFs, or intergenic regions may also contribute to the pathogenesis and outcome of EHV-1. Hence, it is imperative to conduct additional research on ORF30 mutations apart from N752D.

Another unique substitution in Valencia strains was found in regulatory early infected cell protein 22 (ICP22) (ORF65). This mutation involves a change from aspartic acid (D) in the 97P70 strain to asparagine (N) in 21P40 at position 207. This position is located in the acidic-rich area of region 3, which mediates the interaction with immediate early protein 4 (ICP4) and its co-transactivation [41]. Unfortunately, the sequence of ICP22 is only available in a limited number of EHV-1 strains on GenBank, which limits our interpretation of the significance of this mutation in EHV-1 interaction with the host cell and its clinical picture. This is an additional confirmation that the lack of complete EHV-1 genomes minimizes our understanding of the impact of viral factors on the mechanism of EHV-1 infections and the development of EHM [13]. Moreover, to understand the possible impact of mutations in ORF30 and ORF65, as observed in our study, we plan to induce SNP at those two positions in EHV-1 97P70 and determine whether its phenotype changes.

The respiratory mucosal explant model, developed by Vandekerckhove et al. [23], provides a valuable tool as an in vitro model that mimics the in vivo horse situation to have better insights into the pathogenesis of horse respiratory pathogens. This model has been used in previous studies to assess differences in replication between EHV-1 strains, especially between neuropathogenic and abortigenic isolates. In addition, the literature has shown that monocytic cells are the primary carrier cells of EHV-1, which facilitates its dissemination to target organs and has been correlated with the virus's virulence and neuropathogenicity [23, 42–51]. Therefore, in this study, we used the more complex respiratory and vaginal mucosal explant model in parallel to blood monocytes and an RK-13 control cell line to determine the replication behavior of the neuropathogenic 21P40 and abortigenic 97P70 strains.

Upon conducting a comprehensive assessment of the replication and invasion mechanisms of EHV-1 isolates, differences were noted based on the phenotype of the isolate and the organs utilized. The EHV-1 strains, namely neuropathogenic 21P40 and abortigenic 97P70, exhibited varying levels of infection efficiency and kinetics in different mucosal regions and/or cell types. Specifically, 21P40 demonstrated greater efficacy in the upper respiratory mucosa, whereas 97P70 was more efficient at replicating in the vaginal mucosa. This is in agreement with previous studies to compare the neuropathogenic 03P37 and the abortigenic 97P70 strains in nasal [42, 43, 46] and vaginal mucosa [24].

The CD172a^+^ monocytic cells were the predominant infected cells in the epithelium, which confirms the role of monocytic cells as the main target cell and carrier for EHV-1. In our study, some EHV-1 infected cells were neither identified as CD172a^+^ monocytic cells nor CD3^+^ T-lymphocytes. In the epithelium, the explanation could be that most infected cells are epithelial cells. In the lamina propria, unidentified EHV-1 infected cells may be natural killer (NK) or endothelial cells. It is also possible that CD172a and CD3 might be present but at a low level that is not detectable by immunofluorescence.

Cell-associated viremia is a prerequisite for EHV-1 to disseminate to specific organs, including the central nervous system (CNS) and the pregnant uterus. The manifestation of neurologic disease in horses infected with EHV-1 is associated with a long duration of cell-associated viremia at a sustained and elevated level [17, 18, 37]. Past research by our research group indicated that the neuropathogenic 03P37 variant replicated better in blood monocytes than in the abortigenic 97P70 isolate [51, 52]. Our study revealed a comparable finding wherein more infected monocytes were observed with the neurovirulent 21P40 isolate in contrast to the abortigenic 97P70 strain.

The clade 10 neuropathogenic EHV-1 21P40 strain exhibits distinct replication and invasion patterns in the respiratory and vaginal epithelium, despite having minimal genetic alterations (99.96% nucleotide identity and seven SNPs) when compared to the clade 10 abortigenic EHV-1 97P70. Furthermore, the ability of the 21P40 isolate to infect blood monocytes suggests a higher likelihood of inducing neurological complications. The presence of the ORF30 N752D polymorphism cannot be used as a sole marker for the neuropathogenicity of an isolate. Therefore, it is imperative to investigate other potential genetic changes, such as mutations S291I and D207N in ORFs 30 and 65, respectively. Also, we encourage the scientific community to focus on delivering complete high-quality EHV-1 genomic data, including the under-sampled Unique short (Us) region, flanked by repeat regions IR and UR, as they may be associated with alterations in tropism and replication characteristics.

## Supplementary Information

**Additional file 1. Genomic analysis of EHV-1 isolates available in the GenBank repository**. The four single-point amino acid mutations in ORFs 30, 32, 40 and 65 of EHV-1 97P70 and 21P40, in addition to locus 752 of the DNA polymerase (ORF30), were used in the analysis.

## Data Availability

The accession numbers of all strains used in the study are available in the Additional file 1.
