# Correction: Genomic analysis and replication kinetics of the closely related EHV-1 neuropathogenic 21P40 and abortigenic 97P70 strains

**DOI:** 10.1186/s13567-025-01464-5

**Published:** 2025-02-07

**Authors:** Eslam Mohamed, Ines Zarak, Nick Vereecke, Sebastiaan Theuns, Kathlyn Laval, Hans Nauwynck

**Affiliations:** 1 Department of Translational Physiology, Infectiology and Public Health, Faculty of Veterinary Medicine, Ghent University, 9820 Merelbeke, Belgium; 2 Department of Animal Medicine, Faculty of Veterinary Medicine, Benha University, Moshtohor, 13736 Egypt; 3 PathoSense BV, Pastoriestraat 10, 2500 Lier, Belgium

**Correction: Veterinary Research (2025) 56:12** 10.1186/s13567-024-01434-3

Following publication of the original article [1], the authors identified that there is an overlap in Fig. 2.

The original article has been corrected.
