# Supplementary material for: Genomic analysis and replication kinetics of the closely related EHV-1 neuropathogenic 21P40 and abortigenic 97P70 strains
Source: Vet Res. 2025 Jan 13;56:12. doi: 10.1186/s13567-024-01434-3 (PMC11731145; doi:10.1186/s13567-024-01434-3)
Supplement: Supplementary file 1 — Additional file 1. Genomic analysis of EHV-1 isolates available in the GenBank repository. The four single-point amino acid mutations in ORFs 30, 32, 40 and 65 of EHV-1 97P70 and 21P40, in addition to locus 752 of the DNA polymerase (ORF30), were used in the analysis. [file 13567_2024_1434_MOESM1_ESM.pdf]

ORF<br>LOCI	Accession<br>Number	Country	Year	Phenotype	30<br>291	30<br>752	32<br>180	40<br>258	65<br>207
BE/21P40/2021	MW855958	Belgium	2021	Neuropathogenic	I	N	T	C	N
BE/21P41/2021	MW855959	Belgium	2021	Neuropathogenic	I	N	T	C	N
BE/21P43_BD5/2021	MW855960	Belgium	2021	Neuropathogenic	I	N	T	C	N
FR/Valencia1/2021	MW855961	France	2021	Neuropathogenic	I	N	T	C	N
FR/Valencia2/2021	MW855962	France	2021	Neuropathogenic	I	N	T	C	N
H3_Allg_92_21/CH/2021	MZ357402	Switzerland	2021	Neuropathogenic	I	N	T	C	N
YM2019	MT063054	China	2019	Abortigenic	S	N	T	C	D
EHV1_A130	MW316760	Serbia	2018	Abortigenic	-	D	-	-	-
FR-56628	MT968035	France	2018	Neuropathogenic	S	H	-	-	-
BRE/20/2018	MN688630	France	2018	-	S	N	-	-	-
NORM/18/2018	MN688629	France	2018	Abortigenic	S	N	-	-	-
NORM/17/2018	MN688628	France	2018	Abortigenic	S	N	-	-	-
RHONEALPES/16/2018	MN688627	France	2018	Abortigenic	S	N	-	-	-
ILEDEFR/14/2018	MN688626	France	2018	Respiratory	S	D	-	-	-
BELG/12/2017	MN688625	Belgium	2017	Abortigenic	S	N	-	-	-
BELG/10/2016	MN688624	Belgium	2016	Abortigenic	S	N	-	-	-
Hertfordshire/150/2016	KY852346	UK	2016	Abortigenic	S	N	T	C	-
NELLEAQU/9/2015	MN688623	France	2015	-	S	N	-	-	-
NORM/8/2015	MN688622	France	2015	Abortigenic	S	N	-	-	-
EHV1/Meerut/2014	MT077857	India	2014	Abortigenic	S	N	T	C	-
EHV1/Hisar-14/2014	MN912433	India	2014	Abortigenic	S	N	T	C	D
NORM/6/2013	MN688621	France	2013	Abortigenic	S	N	-	-	-
Oxfordshire/207/2013	KU206471	UK	2013	Neuropathogenic	S	N	T	C	-
Oxfordshire/206/2013	KU206470	UK	2013	Neuropathogenic	S	N	T	C	-
Aberdeenshire/84/2013	KU206461	UK	2013	Abortigenic	S	N	-	-	-
Suffolk/41/2013	KU206458	UK	2013	Abortigenic	S	D	-	-	-
Suffolk/45/2013	KU206452	UK	2013	Abortigenic	S	N	-	-	-
Gloucestershire/114/2013	KU206450	UK	2013	Neuropathogenic	S	D	T	C	-
Gloucestershire/108/2013	KU206449	UK	2013	Neuropathogenic	S	D	T	C	-
Gloucestershire/70/2013	KU206448	UK	2013	Neuropathogenic	S	D	T	C	-
Gloucestershire/54/2013	KU206447	UK	2013	Neuropathogenic	S	D	T	C	-
Gloucestershire/77/2013	KU206446	UK	2013	Neuropathogenic	S	D	T	C	-
Suffolk/89/2013	KU206442	UK	2013	Abortigenic	S	N	-	-	-
Suffolk/82/2013	KU206441	UK	2013	Abortigenic	S	N	-	-	-
Cambridgeshire/96/2013	KU206439	UK	2013	Abortigenic	S	N	-	-	-
Suffolk/125/2013	KU206426	UK	2013	Abortigenic	S	N	-	-	-

Suffolk/48/2013	KU206425	UK	2013	Abortigenic	S	N	-	-	-
T970	KC924817	USA	2013	Neuropathogenic	S	D	-	-	-
T956	KC924814	USA	2013	Neuropathogenic	S	D	-	-	-
T75	KC924808	USA	2013	Abortigenic	S	N	-	-	-
T493	KC924800	USA	2013	Abortigenic	S	N	-	-	-
FR-6815	-	France	2013	Abortigenic	-	N	-	-	-
NORM/5/2012	MN688620	France	2012	Abortigenic	S	N	-	-	-
NORM/4/2012	MN688619	France	2012	Abortigenic	S	D	-	-	-
ILEDEFR/3/2012	MN688618	France	2012	Abortigenic	S	N	-	-	-
Lincolnshire/2/2012	KU206475	UK	2012	Abortigenic	S	N	-	-	-
Suffolk/10/2012	KU206474	UK	2012	Abortigenic	S	N	-	-	-
Devonshire/97/2012	KU206469	UK	2012	Neuropathogenic	S	N	T	C	-
Buckinghamshire/24/2012	KU206431	UK	2012	Abortigenic	S	N	-	-	-
KyA	MF975655	USA	2011	Abortigenic	S	D	T	C	D
Oxfordshire/34/2011	KU206457	UK	2011	Abortigenic	S	D	-	-	-
Oxfordshire/31/2011	KU206456	UK	2011	Abortigenic	S	D	-	-	-
Buckinghamshire/93/2011	KU206455	UK	2011	Abortigenic	S	D	-	-	-
Oxfordshire/27/2011	KU206454	UK	2011	Neuropathogenic	S	D	T	C	-
NORM/2/2010	MN688617	France	2010	Abortigenic	S	N	-	-	-
Hertfordshire/188/2010	KU206466	UK	2010	Neuropathogenic	S	D	T	C	-
Buckinghamshire/114/2010	KU206433	UK	2010	Abortigenic	S	N	-	-	-
Norfolk/124/2010	KU206427	UK	2010	Abortigenic	S	N	-	-	-
Cheshire/275/2010	KU206406	UK	2010	Abortigenic	S	N	T	C	-
T953_P210	KR047045	USA	2010	Neuropathogenic	S	D	T	C	D
T953_P135	KR021354	USA	2010	Neuropathogenic	S	D	T	C	D
Hertfordshire/109/2009	KU206476	UK	2009	Abortigenic	S	N	-	-	-
Suffolk/87/2009	KU206443	UK	2009	Abortigenic	S	N	-	-	-
Berkshire/64/2009	KU206438	UK	2009	Abortigenic	S	N	-	-	-
Hampshire/77/2009	KU206419	UK	2009	Abortigenic	S	N	-	-	-
T953_P15	KP975078	USA	2009	Neuropathogenic	S	D	T	C	D
FR-38991	-	France	2009	Neuropathogenic	-	D	-	-	-
Hampshire/1/2008	KU206462	UK	2008	Abortigenic	S	N	-	-	-
3038-07	KT324726	Australia	2007	Neuropathogenic	S	N	T	C	D
3045-07	KT324725	Australia	2007	Abortigenic	S	N	T	C	D
Shropshire/167/2006	KU206405	UK	2006	Abortigenic	S	N	T	C	-
Suffolk/123/2005	KU206480	UK	2005	Abortigenic	S	N	-	-	-
Suffolk/48/2005	KU206430	UK	2005	Respiratory	S	N	-	-	-
Essex/200/2005	KU206411	UK	2005	Abortigenic	S	N	T	C	-
Essex/199/2005	KU206410	UK	2005	Abortigenic	S	N	T	C	-

Bristol/55/2004	KU206424	UK	2004	Abortigenic	S	N	-	-	-
Nottinghamshire/70/2004	KU206420	UK	2004	Abortigenic	S	N	-	-	-
Nottinghamshire/10/2004	KU206404	UK	2004	Abortigenic	S	N	T	C	-
03P37	PP856397*	Belgium	2003	Neuropathogenic	S	D	T	C	D
V592	AY464052	UK	2003	Abortigenic	S	N	T	C	D
United Kingdom/58/2003	KU206444	UK	2003	Neuropathogenic	S	D	T	C	-
Devon/28/2003	KU206440	UK	2003	Respiratory	S	D	-	-	-
2222-03	KT324727	Australia	2003	Abortigenic	S	N	T	C	D
Derbyshire/39/2002	KU206429	UK	2002	Abortigenic	S	N	-	-	-
1966-02	KT324729	Australia	2002	Abortigenic	S	N	T	C	D
2019-02	KT324728	Australia	2002	Abortigenic	S	N	T	C	D
Leicestershire/13/2000	KU206413	UK	2000	Abortigenic	S	N	T	C	-
Yorkshire/114/1999	KU206473	UK	1999	Abortigenic	S	D	-	-	-
Gloucestershire/127/1998	KU206445	UK	1998	Neuropathogenic	S	D	T	C	-
97P70	PP856396*	Belgium	1997	Abortigenic	S	N	I	Y	D
Leicestershire/22/1996	KU206464	UK	1996	Neuropathogenic	S	D	T	C	-
Berkshire/7/1996	KU206463	UK	1996	Abortigenic	S	N	-	-	-
Leicestershire/59/1996	KU206423	UK	1996	Abortigenic	S	N	-	-	-
Suffolk/60/1996	KU206422	UK	1996	Abortigenic	S	N	-	-	-
Wiltshire/40/1996	KU206421	UK	1996	Abortigenic	S	N	-	-	-
Suffolk/16/1996	KU206417	UK	1996	Abortigenic	S	N	-	-	-
Lincolnshire/13/1996	KU206416	UK	1996	Abortigenic	S	N	-	-	-
Lincolnshire/10/1996	KU206412	UK	1996	Abortigenic	S	N	T	C	-
Shropshire/38/1996	KU206407	UK	1996	Abortigenic	S	N	T	C	-
Cambridgeshire/3/1995	KU206432	UK	1995	Abortigenic	S	N	-	-	-
United Kingdom/58/1995	KU206414	UK	1995	Abortigenic	S	N	T	C	-
Suffolk/91/94	KU206479	UK	1994	Abortigenic	S	D	-	-	-
Essex/81/1994	KU206472	UK	1994	Abortigenic	S	N	-	-	-
Suffolk/110/1994	KU206460	UK	1994	Abortigenic	S	D	-	-	-
Kent/43/1994	KU206453	UK	1994	Abortigenic	S	D	-	-	-
United Kingdom/109/1994	KU206434	UK	1994	Abortigenic	S	N	-	-	-
Suffolk/82/1994	KU206409	UK	1994	Respiratory	S	N	T	C	-
1074-94	KT324730	Australia	1994	Abortigenic	S	N	T	C	D
Bristol/2/1993	KU206451	UK	1993	Abortigenic	S	N	-	-	-
Buckinghamshire/9/1993	KU206428	UK	1993	Abortigenic	S	N	-	-	-
Yorkshire/1/1993	KU206418	UK	1993	Abortigenic	S	N	-	-	-
1029-93	KT324731	Australia	1993	Abortigenic	S	N	T	C	D
Ab4	NC_001491	UK	1992	Neuropathogenic	S	D	T	C	D
Kent/177/1991	KU206435	UK	1991	Abortigenic	S	N	-	-	-

Yorkshire/12/1990	KU206437	UK	1990	Abortigenic	S	N	-	-	-
970-90	KT324732	Australia	1990	Abortigenic	S	N	T	C	D
Staffordshire/80/1989	KU206436	UK	1989	Abortigenic	S	N	-	-	-
Shropshire/68/1989	KU206408	UK	1989	Abortigenic	S	N	T	C	-
EHV1_A430	MW316763	Serbia	1987	Abortigenic	-	D	-	-	-
EHV1_A330	MW316762	Serbia	1987	Abortigenic	-	D	-	-	-
EHV1_A230	MW316761	Serbia	1987	Abortigenic	-	D	-	-	-
Suffolk/73/1985	KU206459	UK	1985	Abortigenic	S	D	-	-	-
United Kingdom/106/1985	KU206415	UK	1985	Ocular	S	N	T	C	-
Hong Kong/57/1984	KU206467	Hong Kong	1984	Respiratory	S	N	-	-	-
United Kingdom/32/1982	KU206465	UK	1982	Abortigenic	S	D	T	C	-
717A-82	KT324733	Australia	1982	Neuropathogenic	S	N	T	C	D
438-77	KT324734	Australia	1977	Abortigenic	S	N	T	C	D
NZA-77	KT324724	New Zealand	1977	Abortigenic	S	N	T	C	D
438/77	KF434369	Australia	1977	Abortigenic	S	N	-	-	-
HH1	AB992258	Japan	1970	Abortigenic	S	N	T	C	D
Army 183	KU206477	USA	1941	Respiratory	S	D	-	-	-

- Asterisk at the end of the accession number represents new submissions within the current manuscript.
